# Degenerative Changes in MCP/MTP Joints of Working Horses Without Lameness: Integrating CT-Based Assessment and Synovial Fluid Biomarkers

**DOI:** 10.3390/ani15233392

**Published:** 2025-11-24

**Authors:** Lazar Marković, Ivan Vićić, Mirjana Lazarević Macanović, Jelena Francuski Andrić, Milica Kovačević Filipović, Milena Radaković

**Affiliations:** 1 Department of Equine, Small Animal, Poultry and Wild Animal Diseases, Faculty of Veterinary Medicine, University of Belgrade, Bulevar oslobođenja 18, 11000 Belgrade, Serbia; lazar.markovic@vet.bg.ac.rs; 2 Department of Food Hygiene and Technology, Faculty of Veterinary Medicine, University of Belgrade, Bulevar oslobođenja 18, 11000 Belgrade, Serbia; ivan.vicic@vet.bg.ac.rs; 3 Department of Radiology and Radiation Hygiene, Faculty of Veterinary Medicine, University of Belgrade, Bulevar oslobođenja 18, 11000 Belgrade, Serbia; miramac@vet.bg.ac.rs; 4 Department of Pathophysiology, Faculty of Veterinary Medicine, University of Belgrade, Bulevar oslobođenja 18, 11000 Belgrade, Serbia; jelenaf@vet.bg.ac.rs (J.F.A.); mradakovic@vet.bg.ac.rs (M.R.)

**Keywords:** metacarpo/metatarsophalangeal joints, osteophytes, subchondral sclerosis, computed tomography, Serbian Mountain Horse, oxidative stress biomarkers

## Abstract

Working horses often develop distinct patterns of joint degeneration, yet research in this population remains limited. This study aimed to characterize degenerative changes in the metacarpophalangeal (MCP) and metatarsophalangeal (MTP) joints of Serbian Mountain Horses using computed tomography (CT) and to explore their associations with synovial fluid (SF) biomarkers. A cross-sectional analysis was conducted on 32 MCP/MTP joints from eight clinically sound horses. Postmortem, joints underwent CT imaging, and SF samples were analyzed for viscosity, protein content, total nucleated cell count (TNCC), and oxidative stress biomarkers, along with matrix metalloproteinase (MMP) activity. Subchondral sclerosis was present in all joints, occurring more frequently in horses aged less than nine years. Osteophytosis was observed in two-thirds of joints, and its severity correlated significantly with body weight. Both changes were localized on the medial and lateral condyles and were associated with elevated TNCC, while osteophytosis was linked to increased total oxidant status and oxidative stress index. Relatively low levels of β-hydroxybutyrate appear as a main interactive factor for both changes. No associations were found with MMP activity. These findings indicate that clinically sound working horses commonly exhibit early degenerative joint changes accompanied by mild increases in TNCC and oxidative stress biomarkers.

## Simple Summary

Working horses often experience wear and tear in their joints due to heavy physical activity, but detailed studies on these changes are still rare. Because the early stages of joint damage usually cause no visible pain or lameness, the problem often remains unnoticed until it becomes advanced. This study focused on Serbian Mountain Horses, a local working breed exposed to constant mechanical stress while moving through steep and rough terrain. Using computed tomography (CT) and analysis of synovial fluid, we explored early signs of joint degeneration. The results showed that even horses without visible lameness had subtle structural changes and increased oxidative stress, suggesting that long-term workload can silently trigger early joint damage. Certain synovial fluid indicators, such as total nucleated cell count and oxidative markers, were linked to these structural findings. Identifying such early warning signs may allow detection of joint problems before pain or reduced performance appears. These results improve our understanding of how physical workload affects equine joints and highlight the potential of biomarker-based monitoring to support welfare and longevity in working horses.

## 1. Introduction

Chronic osteoarthritis (OA), commonly referred to as degenerative joint disease (DJD), is characterized by progressive degradation of the articular cartilage, accompanied by structural and functional alterations of the subchondral bone and periarticular soft tissues [1]. The most common bone changes are subchondral sclerosis and osteophytes. Subchondral sclerosis represents increased density and mineralization of the subchondral bone as an adaptive response to chronic mechanical loading within degenerative joint disease [2]. Periarticular osteophytes are widely recognized as a radiographic hallmark of degenerative joint disease, developing through endochondral ossification in response to chronic mechanical stress and joint instability [3,4]. Diagnosing early OA in horses is challenging, as lameness as a sign of pain or gait abnormalities may be intermittent or absent. Lesions are most reliably visualized through arthroscopy, magnetic resonance imaging (MRI), or computed tomography (CT) [5]. On the other hand, these imaging findings do not always fully reflect the pathophysiological processes within the joint. This has led to the exploration of alternative approaches, such as identifying synovial fluid (SF) biomarkers, which enable recognition of OA before evident clinical signs occur.

Oxidative stress and matrix metalloproteinase (MMPs) activity are increasingly recognized as important pathophysiological mechanisms with potential diagnostic implications. Horses with chronic metacarpophalangeal (MCP) and metatarsophalangeal (MTP) OA have increased SF oxidative stress biomarkers [6], as well as those with arthritis secondary to carpal fractures [7]. At the chondrocyte level, increased reactive oxygen species (ROS) can modulate intracellular signaling mechanisms leading to apoptosis, disruption of extracellular matrix turnover, synovial inflammation, and impaired subchondral bone remodeling [8]. Matrix metalloproteinases maintain joint tissue turnover [9], and previous studies reported increased synovial MMP activity in equine OA [10,11]. However, investigations addressing the role of oxidative stress and MMP in early OA without lameness are still scarce.

Working and sport horses experience substantial physical demands due to mechanical stress and repetitive movements, particularly affecting the weight-bearing MCP/MTP joints. High-intensity training is a known contributor to articular cartilage damage and the development of OA [12]. However, working horses endure different types and levels of physical stress compared to sport horses, prompting the question of whether their “lifestyle” influences the distinct pattern of OA development. Despite the economic and cultural significance of working horses in many countries [13], OA research related to this population remains limited.

The Serbian Mountain Horse is a locally adapted working breed that has evolved in the rugged hill and mountainous regions of Serbia. These horses are valued for their endurance and agility in steep, forested terrain that is inaccessible to mechanized equipment [14]. Unfortunately, following their working life, most are slaughtered for the meat industry. The lower limbs, generally excluded from meat processing, represent an underutilized biological resource with significant potential for scientific research.

Using slaughterhouse-derived samples, this study aimed to: (1) characterize the extent and regional distribution of degenerative changes in MCP/MTP joints using CT imaging; and (2) explore the association between imaging-derived findings and SF biomarkers of working horses. To our knowledge, no previous study has integrated oxidative stress biomarker profiling with computed tomography (CT) evaluation of joint changes in the MCP/MTP joints of working horses.

## 2. Materials and Methods

### 2.1. Study Population

This study involved Serbian Mountain Horses that arrived at the slaughterhouse between March and June 2019. The age of each horse was obtained from the horse passport and further verified through dental examination using the dental formula. In Serbia, these types of horses begin working between the ages of two and three and undergo an adaptation period lasting six to twelve months. During the working season, from May to October, these horses carry loads of 100 to 150 kg on their backs. Although precise individual work intensity could not be documented for each horse, all animals originated from similar working conditions, which are characterized by mountain transport and comparable workload.

### 2.2. Clinical Examination

Horses were transported for a maximum of four hours and rested for at least 12 h. The reason for slaughtering was unrelated to this study. After the rest, a general clinical examination was performed, including rectal temperature, heart rate, respiration rate, auscultation of the lungs, heart, and abdomen. The extremities were inspected for the presence of skin lesions, wounds, or edema. If any abnormalities were noted, the horses were excluded from the study. The movement inspection was performed on firm, hard ground, both walking and trotting in a straight line. No flexion tests were performed since horses could only be examined for a short period of time in the slaughterhouse.

Eight Serbian Mountain Horses were selected for further postmortem limb inspection following the outlined criteria: at least 3 years used for pulling/carrying heavy loads, without abnormal clinical findings on clinical examination, and no signs of lameness above grade 2 out of 5 according to the American Association of Equine Practitioners lameness scale (0—no lameness; 1—barely noticeable, intermittent; 2—hard to see at walk/trot but consistent under strain; 3—consistently visible at trot; 4—clearly visible at walk; 5—non–weight-bearing/refuses to move) [15].

### 2.3. Post Mortem CT Examination

Limb imaging was performed using a single-slice CT scanner (Somatom AR Star, Siemens, Healthineers, Erlangen, Germany) with the following parameters: 120 kV, 83 mAs, 2 mm slice thickness, pitch 1, 1 rotation per second, 512 × 517 matrix, and 0.49 mm pixel size. The acquired images were reformatted in sagittal and dorsal planes at 1 mm intervals, generating three images per joint. These images were analyzed using the RadiAnt DICOM Viewer (version 2024.2), with a focus on subchondral sclerosis and osteophytosis. The CT images were evaluated by a single experienced veterinary radiologist (MLM).

For CT image analysis, condyles MCIII/MTIII were divided into: dorsomedial, palmaromedial, dorsolateral, and palmarolateral, while the sagittal metacarpal/metatarsal ridge was divided into dorsal and palmar regions [16].

**Figure 1:**
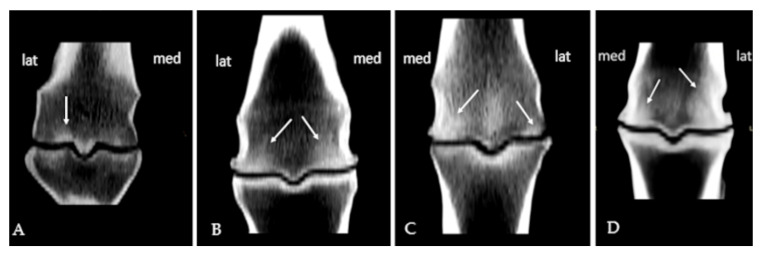
Subchondral sclerosis of the metacarpal condyles of varying grades presented in coronal scan: (**A**) grade 1 on the lateral condyle; (**B**) grade 2 on the lateral and grade 3 on the medial condyle; (**C**) grade 3 on the medial and grade 1 on lateral condyle; (**D**) grade 3 on the medial and grade 4 on lateral condyle (arrows).

**Figure 2:**
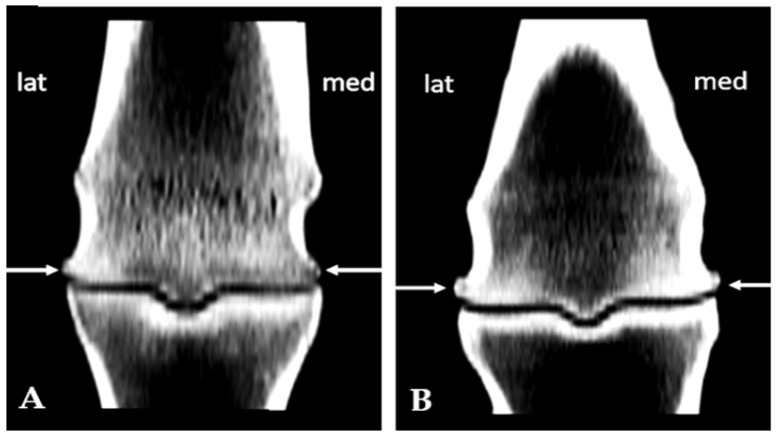
Osteophytes of the metacarpal condyles of varying grades presented in coronal scan: (**A**) grade 1 on the medial and grade 2 on the lateral condyle; (**B**) grade 2 on the medial and grade 3 on the lateral condyle (arrows).

Subchondral sclerosis findings were evaluated on the same previously defined regions using a semiquantitative scale of 0–4. Grade 1 on the scale meant < 10% sclerotic changes; 10–25% sclerotic change was grade 2; 25–50% grade 3, while grade 4 represented more than 50% sclerotic change (Figure 1) [17]. The osteophytosis grades were assigned according to their size using a scale of 0–3, in accordance with the recommended guidelines for assessing OA (Figure 2) [17].

### 2.4. Post Mortem Collection and Routine Analysis of Synovial Fluid

After slaughtering, the legs were harvested from the carpus/tarsus. Within one hour, SF was collected under aseptic conditions by a 21-G needle through the collateral ligament of the proximal sesamoid bone (between the distopalmar/plantar metacarpal/metatarsal condyle and the dorsal part of the lateral proximal sesamoid bone). A minimum of 3 mL of SF was retrieved and placed into 1 mL tubes without anticoagulant.

The viscosity of the SF was semi-qualitatively assessed using a drop of fluid suspended between two fingers and measuring a strand (length expressed in cm) before breakage [18]. The total nucleated cell count (TNCC) was determined by examining synovial smears under a light microscope (Olympus CKS31) using a 100× objective. The total nucleated cell count was calculated using the formula: TNCC per μL = (mean number of cells per field) × (microscope magnification). The mean value was obtained by counting nucleated cells in ten representative microscopic fields. Each slide was independently evaluated by two trained observers (JFA and LM), and the mean values for all numerical variables were calculated. Synovial fluid samples pretreated with hyaluronidase (Sigma H3884, Sigma-Aldrich, St. Louis, MO, USA) were incubated for 20 min at 25 °C, centrifuged at 1400× *g* for 15 min, and the total protein concentration in the supernatant was determined by the biuret method (Elitech, Puteaux, France) using a Technicon RA-KST analyzer (Bayer, Leverkusen, Germany).

### 2.5. Biochemical Analyses of Synovial Fluid

The total oxidant status (TOS) in SF was determined by monitoring the oxidation of ferrous ions to ferric ions under the influence of present oxidases [19]. The ferric ion then forms a colored complex with xylenol orange in an acidic medium, and the color intensity, which is proportional to the total oxidase content, was measured at 560 nm. A standard curve was constructed using hydrogen peroxide solutions.

The total antioxidant capacity (TAC) in SF was determined using the method by Erel [20], applying ABTS^+^ cation as a chromogen. The reaction occurs in an acidic medium, where colorless ABTS is oxidized in the presence of hydrogen peroxide to the intensely colored ABTS^+^ ion. The change in absorbance was monitored spectrophotometrically at 600 nm. For TAC determination, Trolox was used as the standard, prepared in phosphate buffer (pH 7.4). Oxidative stress index (OSI), reflecting the state of redox balance, was calculated as TOS/TAC ratio.

The concentration of total thiols in SF samples was measured using the method outlined by Ellman [21]. In this assay, the DTBN reagent (5,5-dithio-bis-2-nitrobenzoic acid) reacts with aliphatic thiols in a basic medium, resulting in the formation of one mole of p-nitrophenolate anion per mole of thiol. The yellow color intensity of the formed anion was measured spectrophotometrically at 412 nm.

The determination of advanced oxidation protein products (AOPP) levels was performed according to the method previously described by Witko-Sarsat et al. [22]. Synovial samples were diluted with phosphate buffer (pH 7.4) and then mixed with acetic acid and potassium iodide solution. Afterward, absorbance was measured at 340 nm. The concentration of AOPP was expressed in equivalents of chloramine T, which was used to create a standard curve.

Paraoxonase-1 activity (PON-1) was assessed by measuring the increase in absorbance at 412 nm, using 4-nitrophenyl acetate as a substrate [23]. Enzyme activity was calculated using a molar extinction coefficient of 14,000 M^−1^ cm^−1^ and is expressed in units per milliliter.

Ceruloplasmin (CER) concentration in SF was quantified through its p-phenylenediamine oxidase activity, adhering to the Hussein protocol [24].

The concentrations of GSH, TOS, TAC, and CER were determined using an ELISA reader (BioTek Instruments, Winooski, VT, USA), while AOPP concentrations and PON-1 activity were measured using a Cecil 2021 UV/VIS spectrophotometer (Select Science, Bath, UK).

The levels of total proteins, beta-hydroxybutyrate (BHB), uric acid (UA), and cholinesterase activity (CHE) were determined using commercial kits on an automatic biochemical analyzer (Mindray BS-240, Shenzhen, China). Before analysis, each sample was treated with a hyaluronidase in PBS solution (71 g/L).

The activity of gelatinases (MMP–2 and MMP–9) and caseinases (MMP–3 and MMP–13) in ST samples was examined through vertical electrophoresis on polyacrylamide gels [25]. Ten-fold diluted samples were mixed with an equal volume of sample buffer (125 mM Tris, 4% SDS, 20% glycerol, 0.02% bromphenol blue, pH 6.8). Then, each sample was loaded onto an 8% polyacrylamide gel supplemented with 2% gelatin and 1% casein. Electrophoresis was performed in SAS-MX tanks (Helena Laboratories, Beaumont, TX, USA). After electrophoresis, the gels were submerged in a 2% Triton solution and continuously mixed at room temperature for two 30 min intervals. The gels were then incubated at 37 °C in developing buffer (10 mM CaCl_2_, 50 mM Tris, pH 7.5–8) and stained with a 0.25% solution of Coomassie Brilliant Blue G-250 (CBB–G250). After staining, the gels were destained and scanned. Gels were scanned using an EPSON V800 scanner. The presence and activity of MMPs were measured densitometrically using the TotalLab TL120^®^ software, version 1.0.4.0.

### 2.6. Statistical Analysis

All variables with nominal values were included in an univariate logistic regression to determine their individual association with the occurrence of subchondral sclerosis and osteophytosis, as well as the degree of their development on the examined surfaces of the MCP/MTP joints in working horses. Variables with *p* < 0.10 were tested for collinearity and included in a final multivariate logistic regression to determine their relative contribution to the occurrence of subchondral sclerosis. The presence of osteophytes, as well as the degree of osteophytosis and subchondral sclerosis, was significantly associated with only one specific factor, which was presented as the final model. The Hosmer–Lemeshow test was used to evaluate the logistic models (*p* > 0.05). The results are presented as odds ratios (OR) with 95% confidence intervals (CI). Continuous variables were tested for normality of distribution using the Shapiro–Wilk test (*p* > 0.05). A general linear model was used to estimate biochemical parameters of synovial fluid, with age and degree of subchondral sclerosis, as well as live weight and degree of osteophytosis, as fixed factors. The Bonferroni correction test was used for post hoc comparisons. Since the gelatinase showed a non-normal distribution, the non-parametric test (Mann–Whitney U test) was used to compare within-group differences. Statistical analysis was performed using SPSS software version 21 (SPSS Inc., Chicago, IL, USA).

## 3. Results

Eight male Serbian Mountain Horses were included in the study. Their mean age was 9.25 ± 0.36 years, and their average body weight was 515.0 ± 17.06 kg.

### 3.1. Subchondral Sclerosis Presence

Subchondral sclerosis was detected in all MCP/MTP joints in the examined horses. Univariate logistic regression showed that the age of the horses and different localizations on the joint were individually associated with subchondral sclerosis (Supplementary Table S1). Variables with significant associations were entered into the multivariate model to establish their common contributions, with adjustment of their effects (Table 1). A 2.13-fold increased risk for subchondral sclerosis was determined for the working horses younger than 9 years (*p* = 0.02). The probability of subchondral sclerosis appearance was 88% more likely on the condyle compared to the ridge (*p* = 0.0001), and 2.82-fold more likely on the palmar side compared to the dorsal side of the joint. The likelihood of developing subchondral sclerosis grade 2 or greater was higher on the palmar side compared to the dorsal side of the joint (OR = 2.29, 95% CI = 1.15–4.58, *p* = 0.02).

Using a two-factorial model, the individual effect of the age of the working horses and the grade of subchondral sclerosis was found for the examined SF parameters, with the interactive effect only for BHB concentration (Table 2). Horses older than 9 years (*n* = 4) exhibited lower SF viscosity and BHB level than their younger counterparts (*n* = 4) (*p* = 0.02; *p* = 0.002). Among the oxidative stress biomarkers, only uric acid demonstrated a difference between age groups (*p* = 0.04). Higher protein levels were observed in cases with a lower grade of subchondral sclerosis, while AOPP concentrations had an inverse relationship (*p* = 0.02; *p* = 0.04).

### 3.2. Osteophytes Presence

Osteophytosis was detected in 71.0% (95% confidence interval, CI = 52.0–85.8) MCP/MTP joints. The probability of osteophyte presence was significantly higher on the medial and lateral sides compared to the dorso-palmar regions of the MCP/MTP joints (Table 3). With the onset of osteophytosis progression, the likelihood of developing osteophytosis grade 2 was higher on the medial (odds ratio, OR = 10.47, 95% CI = 3.03–36.16, *p* = 0.001) and lateral (OR = 20.08, 95% CI = 5.82–69.32, *p* = 0.001) sides of the joint compared to the dorso-palmar regions. The highest degree of osteophytosis (grade 3) was associated with the body weight of the horses, with the higher probability of its occurrence in horses with ≤500 kg compared to horses weighing more than 500 kg (OR = 5.93, 95% CI = 1.86–18.87, *p* = 0.003).

Considering the weight of the horses and the grade of osteophytosis, an individual effect was determined for the tested SF parameters, with only an interactive effect observed for BHB concentration (Table 4). Total protein and BHB concentrations were associated with weight, being higher in horses with ≤500 kg (*n* = 4) than in heavier horses (*n* = 4) (*p* = 0.09; *p* = 0.03). Differences in osteophytosis grades were evident in TNCC and BHB levels (*p* = 0.05; *p* = 0.04). Oxidative stress biomarkers—TOS and OSI were higher (*p* = 0.01; *p* = 0.02), while the -SH level was lower in horses with higher grades of osteophytosis (*p* = 0.04). Other parameters did not show changes dependent on osteophytosis grades (*p* > 0.05) (Table 4).

### 3.3. Correlation Analysis

Correlation analyses among clinical, imaging-derived, and biochemical parameters are summarized in Table 5 (only significant correlations are reported). Live weight demonstrated a positive correlation with age, and a negative correlation with CHE, BHB, gelatinase activity, and total protein concentration. Live weight had a positive correlation with osteophytosis grade. Age was positively associated with BHB level. The degree of osteophytosis showed a positive correlation with CER and a negative correlation with BHB. Subchondral sclerosis exhibited a positive correlation with TNCC and a negative correlation with BHB.

## 4. Discussion

This study demonstrated that the main degenerative changes observed in the MCP/MTP joints of active working horses without signs of lameness were subchondral sclerosis and osteophytosis. Notably, both changes were related to elevated TNCC, while osteophytosis was also associated with an increase in oxidative stress biomarkers.

Subchondral sclerosis was present in all, whereas osteophytosis in two-thirds of the examined MCP/MTP joints, underscoring that two-thirds of joints had both changes. Their localization corresponded closely, being predominantly situated in the condylar region rather than along the ridge. Moreover, subchondral sclerosis was more pronounced on the palmar aspect/side, a region subjected to significant compressive and shear forces during the stance phase [26,27]. It is known that even horses without lameness have these types and patterns of changes [26,27,28].

### 4.1. Age and Weight Are Linked to Structural Joint Adaptations

Our study showed that younger (≤9 years) horses were at increased risk of developing subchondral sclerosis in certain regions of the MCP/MTP joints. The finding is likely influenced by a sampling bias since the specimens were collected at the slaughterhouse. Although the horses did not display signs of lameness, their removal from work suggests a performance-related issue that could be related to joint or musculoskeletal problems. In our study, body weight correlated positively with the degree of osteophytosis. Seemingly contradictory, a finding shows that grade 3 osteophytes were more prevalent in horses weighing less than 500 kg. We suggest that lighter working horses have higher mechanical stress per unit of the joint cartilage surface and subchondral bone, promoting osteogenic activity and periarticular remodeling. Thus, younger age and lighter weight do not exclude working horses from having structural bone adaptation in the form of osteophytosis.

Synovial fluid protein concentration was lower in heavier than in lighter horses, but always within physiological range [18], and cannot be conclusively attributed to the effects of body weight. This finding warrants further investigation. Furthermore, higher body weight was concurrently associated with reduced BHB levels, suggesting either limited ketogenesis or impaired BHB utilization, thereby diminishing the metabolic resilience of the heavier working horse.

### 4.2. TNCC Emerges as a Sensitive Indicator of Early Degenerative Joint Changes

Horses older than nine years exhibited lower SF viscosity compared to younger individuals. This likely reflects a reduction in both the synthesis and molecular weight of hyaluronic acid, most probably due to mild degenerative changes within the synovial membrane [28,29]. Notably, higher grades of osteophyte formation and subchondral sclerosis were associated with increased TNCC, slightly exceeding the upper physiological range. These elevations occurred despite total protein levels remaining within reference ranges and showed no relationship with age or body weight. This pattern suggests that the changes are primarily associated with degenerative processes linked to chronic joint loading, most likely driven by sustained high-intensity mechanical stress [30,31]. The importance of this finding is highlighted by the positive correlation between higher TNCC values and the extent of macroscopic cartilage lesions and histological evidence of synovial membrane thickening in the same group of horses [32]. This finding, once more, underlines the importance of TNCC as a convenient biomarker of early degenerative joint changes when advanced diagnostic techniques are unavailable.

### 4.3. Osteophytosis Is More Strongly Linked to Oxidative Stress than Subchondral Sclerosis

Excessive mechanical loading has been recognized as a key trigger of oxidative stress [33,34]. Elevated joint pressure may induce transient ischemia, promoting ROS production and their accumulation in SF. Compression enhances SF movement across joint compartments, potentially exposing the entire joint to ROS-mediated damage. Interestingly, the grade of subchondral sclerosis was not associated with oxidative stress except for the increased AOPP. In contrast, horses with advanced osteophytosis exhibited higher TOS and OSI. In horses with advanced osteophytosis, reduced thiol levels—key components of the antioxidant defense system—were also observed, further supporting the presence of heightened oxidative stress in joints displaying both subchondral sclerosis and osteophytosis. Elevated levels of AOPPs in horses with a higher degree of subchondral sclerosis indicate that oxidative protein modification may contribute to joint degeneration. Similarly, Awan et al. [35] reported a positive correlation between circulating AOPP levels and OA severity, supporting their potential as biomarkers in joint disease progression. Other antioxidant parameters remained unchanged, suggesting that pro-oxidative processes dominate under chronic mechanical loading and may drive further joint degeneration. Moreover, relatively low β-hydroxybutyrate (BHB) concentrations were detected in older horses and those with progressive osteophytosis, showing a negative correlation with the severity of osteophytosis. BHB has been reported to mitigate oxidative stress by reducing hydrogen peroxide (H_2_O_2_) levels in human chondrocytes and to alleviate cellular senescence, thereby slowing OA progression [36]. Additionally, aging is associated with altered BHB metabolism and reduced responsiveness of tissues to ketone bodies [37], suggesting that age-related metabolic adaptations may affect BHB production. Hence, lower BHB levels may lead to reduced protective capacity in older working horses and contribute to the development of OA-associated morphological changes. Finally, the observed differences in oxidative stress biomarkers in osteophytosis and their absence in subchondral sclerosis may suggest that osteophytosis represents a more advanced stage of the degenerative process.

It is noteworthy that ceruloplasmin and cholinesterase (CHE)—both acute-phase reactants involved in antioxidant defense—showed no differences in SF between horses with lower and higher grades of degenerative changes. None of the measured parameters indicative of inflammation were elevated, suggesting that inflammation is not a primary pathophysiological mechanism in these early degenerative changes in working horses. Significant elevation in UA level was observed in older horses, but no differences were detected regarding subchondral sclerosis and osteophytosis presence and degree. This implies that the increased concentration of UA, as a non-enzymatic antioxidant, is not a response to oxidative stress in the MCT/MPT joint in older horses, but possibly a result of its age-related decline in renal excretion.

### 4.4. Synovial Fluid MMP Activity Reflects Adaptive Rather than Degenerative Joint Responses

No significant changes in caseinase or gelatinase activity were detected relative to CT-assessed degenerative changes severity. This finding aligns with reports suggesting that synovial MMP activity does not always correlate with structural progression [38,39]. Nevertheless, a positive correlation was found between MMP activity and SF total protein concentrations. This relationship likely reflects a compensatory synovial response aimed at maintaining matrix turnover and joint homeostasis under mechanical stress, rather than indicating pathological degradation or inflammation, as protein concentrations remained within physiological limits. In addition, higher body weight in working horses appears to be related to lower MMP activity. A similar finding was reported in OA patients [40]. Such a pattern may reflect downregulation of catabolic pathways, where sustained stress from pulling heavy loads and bearing higher body weight favors bone remodeling over continuous extracellular matrix degradation, eventually leading to reduced endopeptidase activity.

This study has certain limitations that should be acknowledged. The detailed medical history of the working horses was unknown, including their daily routines, such as duration and intensity of the work, and any previous orthopedic injuries that could provide a better understanding of the issue being examined. The study was conducted on a total of 8 working horses, and 31 MCP/MTP joints were examined. CT analysis included a total of 186 regions of these joints for the presence and degree of subchondral sclerosis and osteophytosis. The relatively limited sample size of the horses may affect the generalizability of the findings.

## 5. Conclusions

These results support the view that chronic mechanical load initiates subclinical oxidative and structural remodeling, progressing toward degenerative joint disease through mechanisms comparable to those seen in sport horses. Taken together, TNCC and oxidative markers may represent practical early indicators of workload-induced joint degeneration in working equids. Future investigations integrating advanced imaging, longitudinal sampling, and controlled workload data are needed to clarify the temporal relationship between oxidative stress, synovial responses, and structural joint changes in working horses.

## Figures and Tables

**Table 1:** Factors associated with the presence of subchondral sclerosis in the working horses.

	B	SE	Wald	OR	95% CI	*p* Value
**Age**						
>9 years	-	-	-	1.00	-	-
≤9 years	0.76	0.34	5.05	2.13	1.10–4.14	**0.02**
**Localization**						
Condyle	-	-	-	1.00	-	-
Ridge	−2.14	0.40	28.27	0.12	0.05–0.26	**0.0001**
**Localization**						
Dorsal	-	-	-	1.00	-	-
Palmar/plantar	1.04	0.34	9.29	2.82	1.45–5.50	**0.002**

B—regression coefficient; SE—standard error of the coefficient; Wald—Wald chi-square statistic used to test the null hypothesis that the coefficient equals zero; OR—odds ratio, representing the exponentiated value of the B; 95% CI—95% confidence interval for the odds ratio; Bold numbers indicate significance of difference (*p*  <  0.05).

**Table 2:** Influence of age and grade of subchondral sclerosis on synovial fluid biomarkers.

Parameter	Age		*p* Value	Subch. Sclerosis Grade		*p* Value	SEM	Interaction
	≤9 Years	>9 Years		≤2	>2			
***n* (number of joints)**	15	16		16	15			
Viscosity (cm)	3.81	2.15	**0.02**	2.48	3.61	0.13	0.46	0.94
TNCC (cells/µL)	1070.67	1338.18	0.27	1001.67	1396.92	0.10	158.34	0.31
Total proteins (g/L)	10.01	9.11	0.42	10.82	8.18	**0.02**	0.74	0.51
CER (mg/dL)	3.70	4.43	0.33	3.81	4.38	0.46	0.52	0.16
CHE (U/L)	562.20	540.37	0.81	575.25	526.93	0.61	65.17	0.35
BHB (mmol/L)	0.25	0.15	**0.002**	0.22	0.18	0.20	0.02	**0.01**
AOPP (µmol/g)	50.61	42.94	0.10	42.20	51.56	**0.05**	3.23	0.76
-SH (mmol/L)	0.14	0.15	0.41	0.14	0.14	0.82	0.01	0.18
PON-1 (U/mL)	2.24	2.36	0.34	2.34	2.24	0.43	0.08	0.07
UA (µmol/L)	60.74	91.52	**0.04**	65.99	88.89	0.18	10.53	0.62
TAC (mmol Trolox Equiv/L)	0.28	0.29	0.79	0.29	0.27	0.08	0.01	0.71
TOS (µM H_2_O_2_ Equiv/L)	119.27	121.65	0.94	133.25	110.18	0.48	20.39	0.40
OSI (AU)	0.39	0.43	0.71	0.49	0.33	0.15	0.06	0.48
Caseinase (AU)	19.83	19.71	0.94	19.39	20.20	0.84	2.90	0.07
Gelatinase (AU)	4.15 (0.14–15.45)	3.16 (0.32–29.16)	0.89	4.35 (0.33–29.16)	1.48 (0.14–20.17)	0.18	-	-

TNCC: total nucleated cell count; CER: ceruloplasmin; CHE: cholinesterase; BHB: β-hydroxybutyrate; AOPP: advanced oxidation protein products; -SH: thiol groups; PON-1: paraoxonase-1; UA: uric acid; TAC: total antioxidant capacity; TOS: total oxidant status; OSI: oxidative stress index; AU: arbitrary units. SEM: standardized error of the mean. Results are expressed as mean except for gelatinase activity, which is presented as median (range). Bold numbers indicate significance of difference (*p*  <  0.05).

**Table 3:** Association of osteophytosis presence and the anatomic region of MCP/MTP joints in examined horses.

	B	SE	Wald	OR	95% CI	*p*-Value
**Localization**						
Dorso-palmar	-	-	-	1.00	-	-
Palmaromedial	2.76	0.59	21.75	15.78	4.95–50.33	**0.001**
Lateromedial	3.32	0.61	29.59	27.87	8.41–92.41	**0.001**

B—regression coefficient; SE—standard error of the coefficient; Wald—Wald chi-square statistic used to test the null hypothesis that the coefficient equals zero; OR—odds ratio, representing the exponentiated value of the B; 95% CI—95% confidence interval for the odds ratio; Bold numbers indicate significance of difference (*p*  <  0.05).

**Table 4:** Influence of the live weight and osteophytosis degree on the synovial fluid biochemical parameters.

Parameter	Live Weight		*p* Value	Osteophytosis Grades		*p* Value	SEM	Interaction
	≤500 kg	>500 kg		<3	3			
***n* (number of joints)**	15	16		20	11			
Viscosity (cm)	3.09	2.87	0.76	2.95	3.17	0.77	0.57	0.31
TNCC (cells/µL)	923.64	1347.69	0.09	975.71	1491.11	**0.05**	148.99	0.59
Total proteins (g/L)	10.88	8.19	**0.01**	9.65	9.45	0.87	0.74	0.20
CER (mg/dL)	3.88	4.27	0.61	3.63	5.11	0.07	0.57	0.55
CHE (U/L)	630.56	466.00	0.06	568.35	521.40	0.64	65.41	0.17
BHB (mmol/L)	0.23	0.16	**0.03**	0.23	0.15	**0.04**	0.02	**0.03**
AOPP (µmol/g)	47.09	47.02	0.99	46.88	46.41	0.93	3.51	0.65
-SH (mmol/L)	0.17	0.15	0.36	0.17	0.13	**0.04**	0.01	0.51
PON-1 (U/mL)	2.35	2.18	0.16	2.24	2.31	0.61	0.07	0.46
UA (µmol/L)	78.28	77.37	0.96	82.44	69.45	0.47	11.27	0.53
TAC (mmol Trolox Equiv/L)	0.28	0.28	0.62	0.28	0.27	0.56	0.01	0.47
TOS (µM H_2_O_2_ Equiv/L)	108.91	91.46	0.47	79.53	140.94	**0.01**	12.03	0.34
OSI (AU)	0.41	0.32	0.32	0.30	0.51	**0.02**	0.04	0.37
Caseinase (AU)	22.41	12.50	**0.01**	20.59	18.83	0.66	2.56	0.86
Gelatinase (AU)	11.16 (0.42–29.16)	1.18 (0.14–6.49)	**0.02**	3.62 (0.14–20.17)	2.11 (0.35–29.16)	0.93	-	-

TNCC: total nucleated cell count; CER: ceruloplasmin; CHE: cholinesterase; BHB: β-hydroxybutyrate; AOPP: advanced oxidation protein products; -SH: thiol groups; PON-1: paraoxonase-1; UA: uric acid; TAC: total antioxidant capacity; TOS: total oxidant status; OSI: oxidative stress index; AU: arbitrary units. SEM: standardized error of the mean. Results are expressed as mean except for gelatinase activity, which is presented as median (range). Bold numbers indicate significance of difference (*p*  <  0.05).

**Table 5:** Significant correlations among clinical, imaging-derived, and biochemical parameters.

Parameters	Coefficient	*p*-Value
Live weight vs. age	0.59	**0.001**
Live weight vs. CHE	−0.48	**0.006**
Live weight vs. BHB	−0.37	**0.04**
Live weight vs. gelatinase	−0.64	**0.001**
Live weight vs. total proteins	−0.60	**0.001**
Live weight vs. osteophytosis grade	0.45	**0.01**
Age vs. BHB	−0.44	**0.01**
Subchondral sclerosis grade vs. TNCC	0.55	**0.005**
Subchondral sclerosis vs. total proteins	−0.44	**0.01**
Subchondral sclerosis presence vs. BHB	−0.72	**0.0001**
Osteophytosis grade vs. BHB	−0.61	**0.001**
Osteophytosis grade vs. CER	0.42	**0.02**

CHE: cholinesterase; BHB: β-hydroxybutyrate; TNCC: total nucleated cell count; CER: ceruloplasmin. Bold numbers indicate significant correlations (*p*  <  0.05).

## Data Availability

The data supporting this study’s findings are available from the corresponding author upon reasonable request.

## Supplementary Materials

The following supporting information can be downloaded at: https://www.mdpi.com/article/10.3390/ani15233392/s1, Table S1: Frequency of subchondral sclerosis in the examined surfaces of the MCP/MTJ joints in working horses.
