# Supplementary material for: Degenerative Changes in MCP/MTP Joints of Working Horses Without Lameness: Integrating CT-Based Assessment and Synovial Fluid Biomarkers
Source: Animals (Basel). 2025 Nov 24;15(23):3392. doi: 10.3390/ani15233392 (PMC12691147; doi:10.3390/ani15233392)
Supplement: Supplementary file 1 [file animals-15-03392-s001.zip › animals-3976053-supplementary.pdf]

Table S1. Frequency of subchondral sclerosis in the examined surfaces of the MCP/MTJ joints in working horses.

	n	Subch. sclerosis %	95% CI	OR*	95% CI	*P* - value
**Age**						**0.04**
> 9 years	96	37.5	28.8-49.0	1.00		
≤ 9 years	90	53.3	42.6-63.7	1.81	1.02-3.25	
**Live weight**						0.79
> 500 kg	96	44.8	34.6-55.3	1.00		
≤ 500 kg	90	45.6	36.3-57.4	1.08	0.61-1.92	
**Localization**						**0.0001**
Condyle	124	59.7	50.9-68.7	1.00		
Ridge	62	16.1	9.0-29.1	0.14	0.07-0.30	
**Localization**						**0.0001**
S_{I}	31	51.6	33.1-69.8	1.00		
S_{II}	31	71.0	52.0-85.8	2.29	0.80-6.53	
S_{III}	31	41.9	24.5-60.9	0.68	0.25-1.84	
S_{IV}	31	74.2	56.6-88.5	2.81	0.97-8.17	
S_{V}	31	12.9	3.6-29.8	0.14	0.04-0.49	
S_{VI}	31	19.4	9.3-40.0	0.26	0.09-0.78	
**Localization**						**0.006**
Dorsal	93	35.5	25.8-46.1	1.00		
Palmar	93	54.8	45.2-66.0	2.29	1.28-4.13	
**Localization**						0.77
Lateral	62	58.1	45.6-71.0	1.00		
Medial	62	61.3	48.1-73.4	1.11	0.54-2.28	
**Localization**						0.54
Front leg	96	47.9	37.6-58.4	1.00		
Rear leg	90	42.2	33.2-54.2	0.84	0.47-1.48	
**Localization**						0.65
LF	48	52.01	37.2-66.7	1.00		
RF	48	43.7	29.5- 58.8	0.72	0.32-1.60	
LH	42	45.2	32.5-63.3	0.84	0.37-1.90	
RH	48	39.6	25.8-54.7	0.60	0.27-1.35	

*OR - odds ratio: S_{I} - dorsomedial zone of the condyle, S_{II} - palmaromedial zone of the condyle, S_{III} - dorsolateral zone of the condyle, S_{IV} - palmarolateral zone of the condyle, S_{V} - dorsal zone of the ridge, S_{VI} - palmar zone of the ridge; LF - left front leg, RF - right front leg, LH - left hind leg, RH - right hind leg.
